# Valproate-induced hyperammonemic encephalopathy enhanced by topiramate and phenobarbitone: A case report and an update

**DOI:** 10.4103/0972-2327.64638

**Published:** 2010

**Authors:** S. Vivekanandan, S. Dinesh Nayak

**Affiliations:** Department of Clinical Biochemistry, Global Hospitals and Health City, Chennai - 600100, India; 1 Neurology, Global Hospitals and Health City, Chennai - 600100, India

**Keywords:** Encephalopathy, hyperammonemia, phenobarbitone, topiramate, valproate

## Abstract

Although sodium valproate (VPA)-induced hepatic encephalopathy is a well-recognized entity, VPA can occasionally produce encephalopathy secondary to hyperammonemia in the presence of normal hepatic function, namely valproate-induced non-hepatic hyperammonemic encephalopathy (VNHE). Known risk factors include therapy with multiple antiepileptic drugs, especially when topiramate is one of the drugs; presence of underlying inborn errors of metabolism; febrile states; and insufficient nutritional intake. We describe a 5-year-old male child who developed VNHE while on polypharmacy with topiramate and phenobarbitone; the child also had poor nutritional intake. The encephalopathy reversed with withdrawal of VPA and treatment with L-carnitine. We emphasize the need for early recognition, investigation, and treatment of this potentially life-threatening condition. We also recommend that VPA, topiramate, and phenobarbitone should not be given in combination.

## Introduction

Sodium valproate (VPA) is an effective first-line antiepileptic drug (AED) that is commonly used in both children and adults with generalized and partial epilepsy syndromes because of its broad spectrum of activity.[1] Although VPA-induced hepatic dysfunction leading to encephalopathy is a well-recognized entity, less commonly the drug can produce encephalopathy of non-hepatic origin by producing hyperammonemia, and is called valproate-induced non-hepatic hyperammonemic encephalopathy (VNHE).[2,3] Delay in recognition of VHE can result in the development of potentially life-threatening complications.[2] Known risk factors for VNHE include AED polypharmacy, febrile conditions, and poor nutritional intake which are thought to deplete L-carnitine levels leading to hyperammonemia).[4-6] Since VPA frequently causes a modest rise in plasma ammonia levels which is asymptomatic, it is important to recognize the symptoms of VNHE promptly and to correlate them with the plasma ammonia levels. In addition, investigations to exclude previously unsuspected underlying metabolic disorders that may be responsible for the hyperammonemia is essential.[7,8]

Although there are several reports of VNHE from the Indian subcontinent,[9-11] we feel it is important to highlight this entity for the benefit of treating physicians. We report a child with VNHE who was on multiple AEDs, including topiramate and phenobarbitone. The encephalopathy reversed following withdrawal of VPA. This emphasizes the importance of rapid diagnosis and treatment in order to prevent neurological damage.

## Case Report

**Figure 1:**
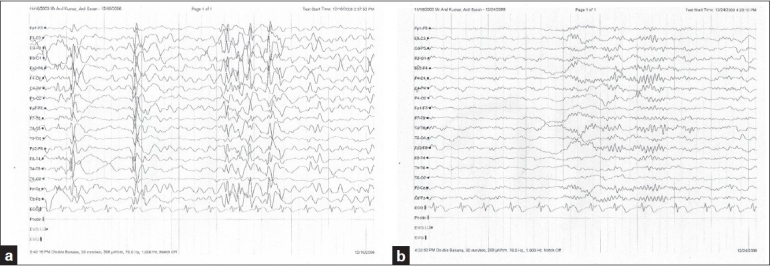
Sedated sleep EEGs: (a) before and (b) 1 week after withdrawal of VPA. (a) Frequent bursts of generalized spike, polyspike, and wave discharges can be seen. Sleep activity is absent. (b) There is marked reduction in epileptiform discharges. Sleep spindles can be seen

A 5-year-old boy was brought to the neurology clinic with history of frequent seizures since the age of 6 months. He was born at term and had normal milestones till 6 months of age, when he developed infantile spasms. He was treated elsewhere with adrenocorticotropin (ACTH) and VPA and, over 12 weeks, the spasms became infrequent. From the age of 3 years, the child started experiencing increased frequency of seizures in the form of sudden bilateral abduction of the arms and extension of trunk that would result in his falling over backwards. Some of the attacks were accompanied by eye blinking and tonic posturing of the limbs for up to 2 min. The frequency had gradually increased from 4–5/day to 10–12/ day over the last 4 months despite the addition of several AEDs. The child became progressively drowsy and lethargic, with alteration of his sleep-wake cycle, poor appetite, and impaired cognition. At presentation, the child was on AED polypharmacy, with daily doses of valproate 30 mg/kg, carbamazepine 25 mg/kg, topiramate 5 mg/kg, levetiracetam 75 mg/kg, phenobarbitone 1.5 mg/kg, lamotrigine, 2.5 mg/kg, and clonazepam 1 mg/day. Magnetic resonance imaging (MRI) revealed right occipital gliosis. Sedated sleep electroencephalogram (EEG) showed very frequent right hemispheric and generalized spike-wave discharges, with absence of normal sleep activity [Figure 1a]. These findings were suggestive of epileptic encephalopathy. Serum ammonia was 117 μmol/l (reference limit: < 35) and blood urea was 7 mg/dl (reference range: 15–45 mg/dl). The rest of the hematological and biochemical (renal, hepatic, and bone) profiles were within normal limits. Hyperammonemia secondary to valproate therapy, enhanced probably by topiramate and phenobarbitone combination was suspected.

We immediately discontinued valproate and started tapering the doses of carbamazepine, topiramate, and phenobarbitone sequentially, with the plan to maintain the patient on lamotrigene and levitiracetam in the long term. A 10-day course of L-carnitine was also given. When reviewed 1 week later, the seizure frequency was only 4–5/day, appetite and sleep–wake cycle returned to normal, and the patient had become more alert and attentive. Plasma ammonia also came down to 43 μmol/l. A repeat EEG showed a significant reduction of epileptiform discharges and a return of normal sleep activity [Figure 1b]. At the end of 3 months the child had gained 4 kg in weight, his seizure frequency had reduced to 2–3/day, and he had a normal blood urea of 25 mg/dl (reference range: 15–45 mg/dl).

## Discussion

Our patient had features of encephalopathy, such as lethargy, alteration of sleep–wake cycle, dulling of sensorium, frequent seizures, and loss of appetite. The presence of significant hyperammonemia with normal liver function tests while on valproate therapy, and the reversal of hyperammonemia after discontinuation of VPA, confirmed the diagnosis of VNHE. It appears that the patient developed VNHE following the addition of topiramate and phenobarbitone (about 6 months earlier) to his regimen of VPA, carbamazepine, levitiracetam, zonisamide, lamotrigine, and clonazepam,. Although AED polypharmacy itself can produce all the symptoms and signs of encephalopathy, such as lethargy, somnolence, ataxia, and cognitive dysfunction, this is unlikely in our patient since the abrupt withdrawal of VPA without simultaneous withdrawal of other AEDs resulted in prompt resolution of the encephalopathic features and improvement in EEG, along with a return of plasma ammonia to near normal levels. It was only over the next few weeks that the other AEDs were withdrawn sequentially. VPA treatment is known to 'unmask' undiagnosed (late-onset) metabolic disorders, including urea cycle defects,[7,8,12] organic acidemias,[8] and other metabolic disorders.[13] This is especially true in children[8,12,13] and, thus, symptomatic hyperammonemia at this age can be a pointer to one of these metabolic disorders.[8] In a metabolic defect, the nonspecific signs and symptoms of hyperammonemia are amplified by precipitating events such as high protein intake, infection, and accidental injury.[8] Urea cycle defects and other metabolic disorders are unlikely to be the cause of hyperammonemia in our patient as there was no previous history of protein intolerance and no reported neonatal, adolescent, or adult deaths in the family. Respiratory alkalosis is a common presenting feature in the initial stages of hyperammonemia caused by urea cycle defects, while ketoacidosis is seen in hyperammonemia caused by an organic acidemia, another common metabolic disorder presenting as symptomatic hyperammonemia.[8] Since the pretest probability of an underlying metabolic disorder was low in our patient (as suggested by the absence of protein intolerance, etc.), we did not do an extensive metabolic workup in our patient. Moreover, since VPA is well known to produce hyperammonemia, especially in the setting of AED polypharmacy, we decided to first withdraw VPA and assess response. The dramatic clinical and EEG improvement noted after withdrawal of VPA justified our approach. We would have proceeded to investigate for underlying metabolic disorders had there been a less dramatic response. Also, virtually all reported cases of VNHE due to metabolic defects have had symptoms of an underlying metabolic disorder that predated the onset of the condition,[7,12,13] whereas our patient had previously been asymptomatic in that respect. We did not check serum VPA levels since there is little correlation between VPA level and clinical effect because of the variable absorption rate and short half-life of the drug.[14] Serum VPA level also poorly correlates with the occurrence of hyperammonemia.[6,11,15]

Ammonia is a by-product of the conversion of amino acids to α-keto acids, primarily via the Krebs-Henseleit urea cycle in the liver, where ammonia is converted to urea for subsequent excretion in the urine.[8] VPA inhibits carbamoyl-phosphate synthetase I, the first enzyme in the urea cycle, thereby hindering the excretion of ammonia and raising plasma ammonia levels. Concomitant use of other AEDs like topiramate[4-6] and phenobarbitone, as well as the use of certain antibiotics like pivmecillinam, may elevate the free (biologically active) fraction of valproate and lead to hyperammonemia.[16] Depletion or reduction of serum L-carnitine levels due to any cause may trigger VNHE.[17,18] VPA enhances urinary excretion of L-carnitine, leading to depletion of blood carnitine stores.[17] Topiramate is also known to deplete L-carnitine. Other risk factors for VNHE include young age, multiple neurological disabilities, and poor nutritional intake.[2,18] The low blood urea levels in our patient suggested poor nutritional intake. Hyperammonemia leads to an increase in the glutamine level in the brain, which produces astrocyte swelling and cerebral edema.[2]

Although there is no consensus on the benefits of L-carnitine supplementation in children on VPA therapy, a Pediatric Neurology Advisory Committee in 1996 has provided the indications for L-carnitine supplementation in childhood epilepsy.[6]

In conclusion, we have the following suggestions to make: 1) Patients and their caregivers must be made aware of the entity of NVHE to facilitate early diagnosis and treatment; 2) AED polypharmacy, especially VPA with topiramate and phenobarbitone, should be avoided, even when seizures are less than satisfactorily controlled; 3) In the setting of hyperammonemia, VPA should be immediately withdrawn; and 4) evaluation for underlying metabolic disorders need only be done when the response to VPA withdrawal is suboptimal or when there are clinical clues to the existence of these disorders; this approach may be cost-effective in our setup.

## References

[1] Marson AG, Al-Kharusi AM, Alwaidh M, Appleton R, Baker GA, Chadwick DW (2007). The SANAD study of effectiveness of valproate, lamotrigine, or topiramate for generalised and unclassifiable epilepsy: An unblinded randomised controlled trial. Lancet.

[2] Duarte J, Macias S, Coria F, Fernandez E, Claveria LE (1993). Valproate induced coma: Case report and literature review. Ann Pharmacother.

[3] Gomceli YB, Kutlu G, Cavdar L, Sanivar F, Inan LE (2007). Different clinical manifestations of hyperammonaemic encephalopathy. Epilepsy Behav.

[4] Zaccara G, Paganini M, Campostrini R, Moroni F, Valenza T, Messori A (1985). Effect of associated antiepileptic treatment on valproate-induced hyperammonemia. Ther Drug Moni.

[5] Hamer HM, Knake S, Schomburg U, Rosenow F (2000). Valproate-induced hyperammonemic encephalopathy in the presence of topiramate. Neurology.

[6] Cheung E, Wong V, Fung CW (2005). Topiramate-valproate-induced hyperammonemic encephalopathy syndrome: Case report. J Child Neurol.

[7] Oechsner M, Steen C, Sturenberg HJ, Kohlschütter A (1998). Hyperammonemic encephalopathy after initiation of valproate therapy in unrecognized ornithine transcarbamylase deficiency. J Neurol Neurosurg Psychiatry.

[8] Scriver CR, Beaudet al, Sly Ws, Valle D (2001). The metabolic and molecular bases of inherited disease.

[9] Panda S, Radhakrishnan K (2004). Two cases of valproate-induced hyperammonemic encephalopathy without hepatic failure. J Assoc Physicians India.

[10] Rath A, Naryanan TJ, Chowdhary GV, Murthy JM (2005). Valproate-induced hyperammonemic encephalopathy with normal liver function. Neurol India.

[11] Mehndiratta MM, Mehndiratta P, Phul P, Garg S (2008). Valproate induced non hepatic hyperammonaemic encephalopathy (VNHE): A study from tertiary care referral university hospital, north India. J Pak Med Assoc.

[12] Heron B, Gautier A, Dulac O, Ponsot G (1993). Biotinidase deficiency: Progressive encephalopathy curable with biotin. Arch Fr Pediatr.

[13] Walser M (1986). Role of urea production, ammonium excretion, and amino acid oxidation in acid base balance. Am J Physiol.

[14] Lundberg R, Nergadh A, Borrus IO (1982). Plasma concentration of valproate drug maintenance therapy in epileptic children. J Neurol.

[15] DeWolfe JL, Knowlton RC, Beasley MT, Cofield S, Faught E, Limdi NA (2009). Hyperammonemia following intravenous valpraote loading. Epilepsy Res.

[16] Lokrantz CM, Eriksson B, Rosén I, Asztely F (2004). Hyperammonemic encephalopathy induced by a combination of valproate and pivmecillinam. Acta Neurol Scand.

[17] Beversdorf C, Allen C, Nordgren R (1996). Valproate induced encephalopathy treated with L-Carnitine in an adult. J Neurol Neurosurg Psychiatry.

[18] De Vivo DC, Bohan TP, Coulter DL, Dreifuss FE, Greenwood RS, Nordli DR (1998). L-Carnitine supplementation in childhood epilepsy: Current perspectives. Epilepsia.

